# Geographic reach and nutritional quality of foods available from mobile online food delivery service applications: novel opportunities for retail food environment surveillance

**DOI:** 10.1186/s12889-021-10489-2

**Published:** 2021-03-06

**Authors:** Keshbir Brar, Leia Michelle Minaker

**Affiliations:** 1 School of Public Health and Health Systems, University of Waterloo, 200 University Ave W, Waterloo, ON N2L 3G1 Canada; 2 School of Planning, University of Waterloo, 200 University Ave W, Environment 3 Rm. 3239, Waterloo, ON N2L 3G1 Canada

**Keywords:** Food environment, Food delivery, OFDS, Mobile app, O2O

## Abstract

**Background:**

Online Food Delivery Services (OFDS) have rapidly expanded in North America, but their implications for geographic access to food and potential dietary outcomes of their use are poorly understood. The purpose of this paper is to examine the extent to which OFDS may geographically expand retail food environments. A secondary objective is to evaluate the healthfulness of foods available on mobile OFDS in a large Canadian city using the Healthy Eating Index-2015 (HEI-2015).

**Methods:**

Retailers’ distance from delivery location was assessed on a large ODFS platform using 24 randomly selected urban postal codes in Ontario, Canada (*n* = 480 retailers). Distance to the first 10 and the last 10 listed retailers in each postal code was examined in relation to a) city population, b) city population density, and c) whether retailers appeared first or last. Second, to determine the healthfulness of food items available, menus of twelve retailers (*n* = 759 menu items) from four popular OFDS platforms available in Mississauga, Ontario, were coded using the Food and Nutrient Database for Dietary Studies-2015, and Food Patterns Equivalents Database-2015. Coded items were used to derive HEI-2015 scores.

**Results:**

Delivery distances from the sample of postal codes in Ontario ranged from 0.3 km to 9.4 km (mean 3.7 km), and the total number of retailers available to each postal code ranged from 33 to 472. Substantial, positive correlations existed between total number of retailers available and both city population (r = 0.71), and population density (r = 0.51). HEI-2015 scores for retailers’ full menus were typically low, and ranged from 19.95 to 50.78 out of 100.

**Conclusions:**

OFDS substantially increases geographic access to foods prepared away from home (by up to 9 km and 472 restaurants). Food offerings on OFDS applications do not meet healthy eating recommendations. Given the projected continued rapid expansion of OFDS, particularly in the midst of a global pandemic, surveillance and future research on OFDS and population dietary health is warranted.

## Background

Global food activity trends indicated populations are consuming more processed foods and food away from home (FAFH), resulting in poor diet quality and an increase in sedentary behaviours [1, 2]. FAFH is defined as food prepared outside the home, and includes food that is eaten out, ordered-in and/or delivered [3]. FAFH comprises prepared, typically energy-dense foods with low nutritional value, and its consumption has been associated with an increased risk for non-communicable diseases such as obesity, cholesterol and diabetes, among others [4, 5]. All of these non-communicable diseases significantly contribute to a country’s disease burden, and subsequent healthcare expenditure [3].

The consumption of FAFH may be influenced by the food environment, as it determines geographic access to food, and influences food behaviours via external cues from the environment [6, 7]. Food environments are typically classified based on geographical access to different types of food sources (e.g., supermarkets or fast food outlets) in a given geographical area [8]. Historically, this has included measuring the proximity to, and/or density of supermarkets, convenience stores, fast-food retailers and grocery stores in a particular neighbourhood [8]. Within Canada, obesogenic food environments or “food swamps” are prominent, especially in low income areas [9]. Food swamps are categorized by a high density of fast-food outlets and convenience stores (i.e. sources typically understood to provide less nutritious foods), and may promote the consumption of FAFH, subsequently leading to poor health outcomes [9].

Over the past decade in North America, a new FAFH source has quickly risen to prominence: Online Food Delivery Services (OFDS). While OFDS are common in East Asian countries like China, their emergence in a North American setting is relatively new, and their impact on the food environment and human health is not well understood [10]. In China, men, college students, white-collar workers, those of Asian heritage, and individuals with higher socio-economic status seem to use OFDS more heavily than their counterparts [10]. Additionally, a lack of cooking skills, and a high comfort level with technology is associated with higher likelihood of OFDS use [10, 11]. Extant food environment research fails to account for the impact mobile OFDS could have on geographic food access and on population-level diets. Studies from China indicate that OFDS have the potential to widen food access by more than 10 km, which is beyond what is traditionally assessed to determine a food environment [10], but to date, no studies have examined the extent to which OFDS might change geographic retail food access in a North American context. This is an important gap, given that the UBS investment bank has projected that global OFDS sales will grow from $35 billion to $365 billion by 2035 [12].

Research gaps related to how OFDS changes access to food and dietary health are important, given that OFDS are predicted to rapidly expand in the coming decades. The overarching goal of this research is to provide a foundation for ultimately understanding how OFDS affects dietary health at a population level. The primary objective of this research is to therefore examine the extent to which OFDS may geographically expand retail food environments. A secondary objective is to evaluate the healthfulness of foods and beverages available on mobile OFDS, given that no studies to date do so, and while diet quality of fast-food menu offerings is consistently poor [13], it is currently unknown whether OFDS offerings differ from brick-and-mortar offerings in terms of diet quality. To achieve objective one, we examine minimum, maximum, and mean delivery distances from 24 randomly selected urban postal codes in Ontario. Second, we use the HEI-2015 to assess diet quality of menu offerings of OFDS restaurants serving Mississauga, Ontario.

## Methods

### Objective 1: OFDS and geographic food access

To address objective one and examine how OFDS applications may geographically expand retail food environments, the distance of each retailer from the delivery location was analyzed using the DoorDash application (hereafter, app). DoorDash was selected because at the time of data collection it had the largest service range of OFDS applications in Ontario. At the time, DoorDash serviced 850 cities across North America, in comparison to UberEats which serviced over 50 cities in 13 countries, and SkiptheDishes, which serviced over 100 cities across Canada.

We randomly selected 24 postal codes with access to DoorDash in the province of Ontario (Canada’s most populous province). First, we obtained the most recent Ontario Postal Code Conversion File from Statistics Canada (2017) [14]. Postal code data was cleaned by removing rural postal codes, since DoorDash did not service these areas at the time of data collection. The random number generator in Microsoft Excel was used to generate a list of random numbers between 0 and 1, which were assigned to the list of 401,253 postal codes in Ontario. The random numbers and associated postal codes were sorted in ascending order, and 50 urban postal codes were selected as a starting point for checking whether DoorDash delivered to these areas. From the list of 50 randomly selected postal codes, the DoorDash app was used to determine whether that particular postal code area was serviced until a total of 24 postal codes with DoorDash service were reached.

Given that the algorithms for the order in which retailers are displayed on the app are proprietary in nature, and that it is reasonable to suspect that display order may influence retailer selection, we selected the first 10 retailers that appeared on the app, and the last 10 retailers that appeared on the app for each of the 24 postal codes. Therefore, we identified 20 retailers per postal code, resulting in a dataset of 480 retailers, each of which was associated with a postal code and the distance between the postal code and the retailer (distance was specified by the DoorDash app). Additionally, we collected the total number of retailers available on DoorDash for each postal code. For each postal code (based on the 20 retailers per postal code), we estimated the minimum retailer distance (km), maximum retailer distance (km), and mean retailer distance (km). We also estimated the minimum, maximum, and mean retailer distances for the first 10 retailers displayed as well as the last 10 retailers displayed. Finally, we used 2016 Canadian census data to determine the population size and population density of the cities in which each of the postal codes were located to further explore the context of OFDS and geographical food access. Specifically, we examined Pearson correlations between the total number of retailers available to a given postal code, and the population size or population density using the = CORREL command in MS Excel. We also examined correlations between the total number of retailers available and travel distances, and further explored how population density and mean distance to the first 10 restaurants displayed vs. the last 10 restaurants displayed compared.

### Objective 2: examining healthfulness of OFDS menus

The following methods sections describe how we addressed objective two, specifically by examining the diet quality of menus of OFDS retailers serving the city of Mississauga Ontario.

#### Identifying OFDS apps

**Fig. 1:**
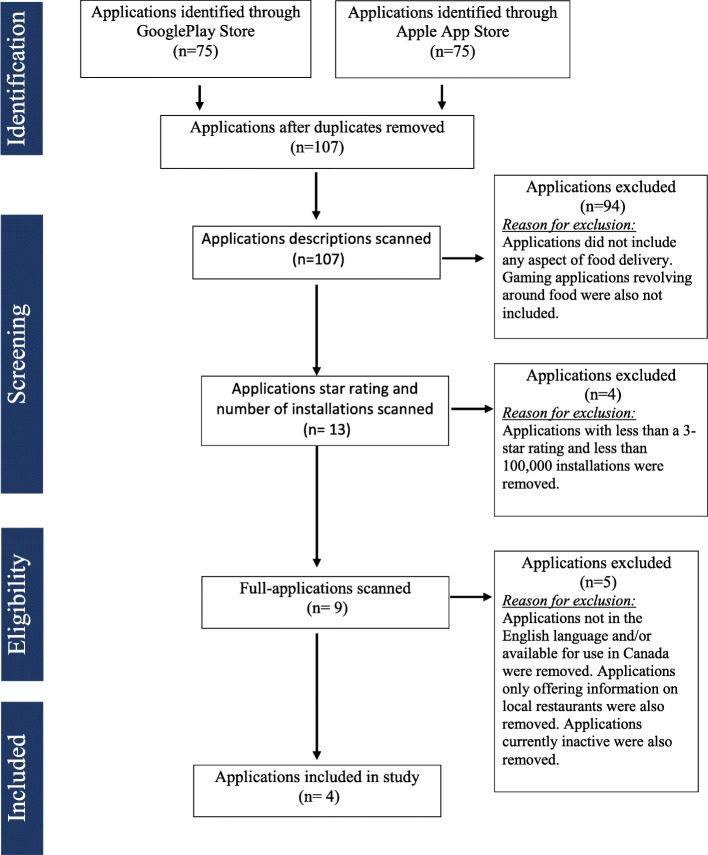
OFDS Application selection process

Figure 1 provides an overview of the OFDS application selection process. To start, GooglePlay and the Apple app store (hereafter, App store) were searched in January 2019 for mobile OFDS applications available for free public use within Mississauga, Ontario, in the English language. Three different searches were conducted on GooglePlay and the App store using the following terms: “food delivery apps”, “food take out apps”, “online food delivery service”. The first twenty-five returns for each search were included in the study for further screening.

GooglePlay and the App store were selected as a majority of mobile users either use an Android operating system (70%) or an iOS operating system (28.5%) [15]. Along with these operating systems, specific bundled applications like GooglePlay and App store are included to allow user to downloaded other applications [15]. These two operating systems currently dominate the mobile market in North America and thus it is a reasonable assumption that most users downloading OFDS applications are doing so through these operating systems.

#### Inclusion and exclusion criteria

Applications were included if they were in English, available for free public use in Mississauga, available on both GooglePlay and App Store, and had a minimum 3-star rating or over one million installs. A 3-star rating was used as a standard as anything lower may be considered a deterrent for people to actually download the application. Applications were excluded if they were food delivery games, food trackers, food databases, targeted applications for particular dietary ailments, and/or did not offer food delivery via application.

#### Application selection

A total of 150 applications emerged from the search: 75 from GooglePlay and 75 from the App store. Upon duplication removal, 107 applications remained. These applications titles and descriptions were further assessed based on inclusion and exclusion criteria. After the title and description scan, 13 applications were scanned based on star rating and number of installations. Nine applications met inclusion criteria at this stage, all of which were downloaded and fully scanned. Of the nine applications, four met all inclusion criteria and were included in the study: UberEats, DoorDash, SkiptheDishes, and Foodora.

### Measuring dietary quality of OFDS retailer menus

In order to measure the healthfulness of food available on OFDS, the Healthy Eating Index – 2015 (HEI-2015) was used [16]. Although the HEI-2015 is typically used to assess individual diets, it can be used to assess the community food environment – places where individuals acquire food. For instance, research conducted by Kirkpatrick and colleagues, assessed the healthfulness of menus for five major fast food outlets using the HEI-2005 [13]. For this reason, it can be used to assess the healthfulness of menus for retailers available on UberEats, DoorDash, SkiptheDishes, and Foodora. Of note, although there is a Canadian counterpart HEI-C (2010) [17], we elected to use the HEI-2015 for two reasons. First, as part of deriving diet quality scores, menu items coded using the Food and Nutrient Database for Dietary Studies (2015) needed to be linked to the USDA’s Food Patterns Equivalents Database (2015) to determine the value of other dietary constituents (e.g., fruits, vegetables, lean meats). Unfortunately, the Canadian counterpart database did not link appropriately with the food codes for the HEI-C. Second, the Food and Nutrient Database for Dietary Studies (2015) and the linkable USDA’s Food Patterns Equivalents Database (2015) were the most recent version of both databases available at the time of the study, which helped to ensure matches between database foods and current menu foods available.

The HEI-2015 is designed to align with the 2015–2020 Dietary Guidelines for Americans [16]. As shown in Table 1, the HEI-2015 is comprised of 13 components that sum to a total maximum of 100 points [16]. Each component is scored on a density basis out of 1000 cal, with the exception of Fatty Acids, which is a ratio of unsaturated fatty acids to saturated fatty acids [16]. Higher scores are assigned when higher quantities of food are present for each category, with the exception of refined grains, sodium, added sugar, and saturated fats, where lower quantities are assigned a higher score. In short, a higher HEI-2015 total score means the set of food under consideration is healthier in relation to healthy eating recommendations.

**Table 1:** HEI-2015 Dietary Components, Constituents, and Scoring Standards

Component (units)	Dietary Constituents	Maximum Score	Standard for Maximum(***Standard for Minimum, if Moderation Component***)
**From Food Patterns Equivalence Database (or other food-based database)**			
**Total Fruits (**cup eq.)	Total Fruit	5	≥0.8 cup eq. per 1000 kcal
**Whole Fruits (**cup eq.)	Citrus, Melons, Berries + Other Intact Fruits	5	≥0.4 cup eq. per 1000 kcal
**Total Vegetables (**cup eq.)	Total Vegetables + Legumes (Beans and Peas) in cup equivalents	5	≥1.1 cup eq. per 1000 kcal
**Greens and Beans (**cup eq.)	Dark Green Vegetables + Legumes (Beans and Peas) in cup equivalents	5	≥0.2 cup eq. per 1000 kcal
**Whole Grains (**oz. eq.)	Whole Grains	10	≥1.5 oz. eq. per 1000 kcal
**Dairy (**cup eq.)	Total Dairy	10	≥1.3 cup eq. per 1000 kcal
**Total Protein Foods (**oz. eq.)	Total Meat, Poultry, and Seafood (including organ meats and cured meats) + Eggs + Nuts and Seeds + Soy + Legumes (Beans and Peas) in oz. equivalents	5	≥2.5 oz. eq. per 1000 kcal
**Seafood and Plant Proteins (**oz. eq.)	Seafood (high in n-3) + Seafood (low in n-3) + Soy + Nuts and Seeds + Legumes (Beans and Peas) in oz. equivalents	5	≥0.8 oz. eq. per 1000 kcal
**Refined Grains (**oz. eq.)	Refined Grains	10	≤1.8 oz. eq. per 1000 kcal(*≥4.3 oz eq. per 1000 kcal*)
**Added Sugars (**tsp. eq.)	Added Sugars	10	≤6.5% of energy (*≥26% of energy*)
**From FNDDS (or other nutrient database)**			
**Fatty Acids (g)**	(Total Monounsaturated Fatty Acids + Total Polyunsaturated Fatty Acids)/Total Saturated Fatty Acids	10	(MUFAs + PUFAs) /SFAs≥2.5((MUFAs + PUFAs)/SFAs≤1.2)
**Sodium (mg)**	Sodium	10	≤1.1 g per 1000 kcal (*≥2.0 g per 1000 kcal*)
**Saturated Fats (g)**	Total Saturated Fatty Acids	10	≤8% of energy (*≥16% of energy*)
**Energy (kcal)**	Total Energy	N/A	N/A

#### Identifying the set of foods under consideration

OFDS applications require an address to be entered before retailers appear to users. For this study, the address entered for all applications was a unique postal code in Mississauga, ON. Mississauga is a city of over 800,000 within the Greater Toronto Area. The analysis focused on assessing the full-menu of the first three retailers that appeared on each OFDS to users. A total of 12 retailer’s full menus were assessed. We elected not to incorporate children’s menus for two reasons: First, of the limited evidence that exists, some indicates that college students and individuals with higher socio-economic status are more likely to use OFDS [10]. Second, the OFDS apps required users to be at least 18 years of age. It is important to note that it was beyond the scope of the current study to compare foods available via OFDS platforms vs. items available in brick-and-mortar retailers, and only the items available on OFDS platforms were assessed.

#### Determining the amount of relevant dietary constituent in the set of foods

##### Operationalizing the set of foods to be analysed for each retailer

Menu items from each retailer were entered into an excel document to ensure only items available on the OFDS were assessed. All menu items were considered unique and were only counted once. Items that were part of a combo were counted separately, however if they were captured in a previous menu item it would not be listed again. For instance, a specific burger would only be counted once, and if it appeared again in a meal or combo it was not counted again. Additionally, if a menu item came in a variety of different sizes, each size of the menu item was counted as a unique menu item. However, beverages that were offered both hot or cold (i.e. hot vanilla bean latte versus cold vanilla bean latte) were treated as identical as they did not have any caloric or nutritional differences. Calorie-free items such as diet soft drinks were also included as they may contribute to the HEI-2015 sodium component.

##### Coding menu items

Menu items were coded using the United States Department of Agriculture (USDA) Food and Nutrient Database for Dietary Studies (FNDDS) 2015. FNDDS foods that could be directly assigned to a Food Patterns (FP) component, were assigned based on accuracy of food description to the actual item. Items that could not be assigned to an FP component directly were disaggregated into their ingredients and appropriately matched to an FP component thereafter. When disaggregating food items into their ingredients, the following assumptions were made for each retailer:

- No extra items were added to menu items (i.e. extra mayo);
- No substitutions were made (i.e. rice instead of noodles);
- Assumed white rice was offered (rather than brown), unless otherwise specified on the menu;
- For items that required extra toppings, it was assumed all toppings were added;
- For pizzas, it was assumed regular (white) pizza dough was used unless otherwise specified on the menu (i.e. whole wheat base);
- For wraps it was assumed a white pita was used, unless otherwise specified on the menu.

Items that were considered to be non-FP components (i.e. black coffee without added sugar) based on FNDDS criteria, were labelled as non-FP. Typically, non-FP components included foods and beverages that contain a substantial proportion of ingredients that are not conventional FP components [18]. The retailers’ online menus were used to retrieve information on energy, saturated fat, trans fat and sodium. This included McDonalds, Little Caesars, A&W, Hero Certified Burgers, Quik Chik, Pizza Pizza, Edo Japan, and Milestones. However, for retailers with no dietary information available online or missing information, the FNDDS was used to fill in the gaps. This included Halal Guys, Supermoon Japanese Style Cheesecakes, Lazeez Shawarma, Fattoush Mediterranean.

##### Linking databases

Once coded, the energy reported in the retailer’s nutrient information was compared with the FNDDS energy value for all items to ensure equivalency. The FNDDS codes were then used to link to the USDA’S Food Patterns Equivalents Database 2015 to determine the value of other dietary constituents such as fruits, vegetables, lean meat, dairy, etc.

##### Derive pertinent ratios of dietary constituents to energy and score each HEI-2015 component

In order to derive pertinent ratios of dietary constituents, the population ratio method was used, as per Kirkpatrick and colleagues’ methods [13]. For each HEI component, dietary constituents of the entire menu are summed together to provide a total HEI component score. This value is then used to determine dietary constituents to 1000 kcal of energy, with the exception of saturated fatty acids and added sugars which are converted to kcal by multiplying by 9 and 16 respectively. Saturated fatty acids and added sugars are expressed as a percent of calorie basis. Ratios were then derived by taking each dietary constituent and dividing by the total energy. This ratio was compared to the HEI-2015 Standards to assign a score. All of these calculations were completed by a SAS algorithm provided by National Cancer Institute.

## Results

### Objective 1: OFDS and geographic food access

The distance between the “delivery location” (postal code) and retailer ranged from 0.3 km to 9.4 km, with a mean of 3.7 km. The mean distance of the 10 retailers that appeared first was 3.2 km (range 0.3 km to 7.9 km), while the mean distance of the last 10 retailers was 4.6 km (range 0.5 km to 9.4 km). The total number of retailers available for each postal code ranged from 33 to 472.

Of the 24 randomly selected urban postal codes in Ontario, 13 unique cities were represented. Although population size and population density were assessed at the city level from census data, the number of retailers ranged substantially even between postal codes within the same city. For example, one postal code in Brampton, Ontario (population approximately 594,000 in 2016) had 39 retailers available on DoorDash, while another postal code in Brampton had 162 retailers available. Similarly, in Mississauga, Ontario (population approximately 722,000 in 2016), one postal code had 49 retailers available while another in Mississauga had 181 retailers available.

In terms of the correlations we explored, the correlation between city population size and the total number of retailers was 0.71, and the correlation between population density and the total number of retailers was 0.51. Somewhat surprisingly, there was only a weak negative correlation between the total number of retailers and the mean travel distance (r = − 0.16), and an even lower correlation between the total number of retailers and minimum travel distance (r = 0.07).

**Fig. 2:**
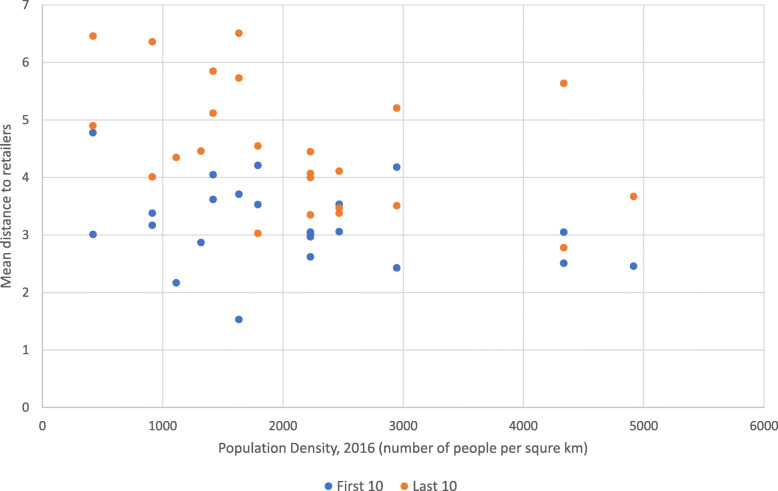
Mean distance to retailers by Population Density (First 10 displayed and Last 10 displayed retailers on DoorDash, 2019)

Figure 2 shows the mean distance to retailers by population density of the first 10 and last 10 retailers displayed on DoorDash. The overall correlation between population density and mean distance to retailers was expectedly negative and medium in strength (r = − 0.48). The correlation between population density and the mean distance of the first 10 retailers displayed on the app was medium and negative (r = − 0.30), while the correlation between population density and the mean distance of the last 10 retailers was slightly stronger at − 0.43. In other words, for both the first 10 and the last 10 retailers displayed, as population density of the postal code city increased, mean distance to retailers decreased.

### Objective 2: examining healthfulness of OFDS menus

A total of 759 menu items were assessed across 12 menus from the 12 OFDS retailers selected. Of the 759 menu items, 97 (12.8%, range 4–18 per menu) were appetizers, 315 (41.5%, range 14–36 per menu) were entrees, 67 (8.8%, range 1–47 per menu) were desserts, 257 (32.5%, range 3–72 per menu) were beverages, and 33 (4.3%, range 4–12 per menu) were sauces and dressings.

Retailers ranged from traditional fast-food to more niche shops (i.e. falafel, Japanese food). McDonalds, A&W, Hero Certified Burgers, and Quik Chik menus were made up of typical fast-food items such as burgers, fries, sodas, and fried chicken. Lazeez Shawarma, Fattoush Mediterranean Grill and the Halal Guys menu items reflected middle eastern food choices such as falafels and flatbreads. Little Caesars and Pizza Pizza menu items included a variety of different pizzas. Edo Japan’s menu items included Japanese style soups, broths and bowls. Milestones menu items reflected typical sit-down menu items including main dishes with salmon and salads with fruits and vegetables. Supermoon Japanese Style Cheesecakes menu items were unique in the sense that they strictly offered Japanese style pastries and bubble tea drinks.

Total HEI-2015 scores out of 100 for the full menus of these retailers ranged from 19.95 (Quik Chik) to 50.78 (Pizza Pizza; Table 2). The components that typically scored well for most retailers include: Total Vegetables, Greens and Beans, Dairy, Total Protein Fruits, Seafood and Plant Proteins. Components that generally scored poorly included: Total Fruit, Whole Fruit, Whole Grains, Added Sugars, Saturated Fats, Refined Grains, Sodium, Fatty Acid Ratio.

**Table 2:** HEI-2005 component and total scores for full menus, by retailer and OFDS

	Ubereats			DoorDash			SkiptheDishes			Foodora		
Component (maximum score)	McDonalds	Lazeez Shawarma	Little Caesars	A&W	Hero Certified Burgers	Quik Chik	Pizza Pizza	Edo Japan	Fattoush Mediterranean Grill	The Halal Guys	Milestones	Supermoon Japanese Style Cheesecakes
Total Vegetables [5]	0.94	5	2.26	5	5	1.86	4.84	5	5	5	5	0
Greens and Beans [5]	0.51	5	0	0	0	0.73	3.42	5	5	5	5	0
Total Fruit [5]	0.69	0.23	0.27	0.64	0.17	0.97	0.16	0.12	0.12	0.91	1.3	0
Whole Fruit [5]	1.38	0.45	0.55	1.28	0.34	1.95	0.32	0.24	0.24	1.82	2.6	0
Whole Grains [10]	0.44	0.07	0	0.18	0	0.43	0	3.85	3.85	0	2.72	0
Dairy [10]	10	4.42	6.93	10	10	4.08	10	0.11	0.11	1.04	10	3.13
Total Protein Foods [5]	2.37	5	2.97	5	5	5	3.69	5	5	5	5	0.94
Seafood and Plant Proteins [5]	2.9	3.67	0	5	1.78	4.93	0.6	5	5	5	5	0
Fatty Acid Ratio [10]	0	0	0	5.89	0	0	8.97	0	0	0	0	0
Sodium [10]	0	1.38	2.22	3.02	2.68	0	0	1.03	1.02	6.76	0	10
Refined Grains [10]	10	0	4.84	0	3.61	0	4.82	10	10	9.99	9.58	7.36
Saturated Fats [10]	0	0	0	0	0	0	10	0	0	0	0	0
Added Sugars [10]	0	0.91	0	0	0	0	3.96	0	0	9.09	0	0
**Total HEI-2015 Score**	**29.23**	**26.13**	**20.04**	**36.01**	**28.58**	**19.95**	**50.78**	**35.35**	**35.34**	**49.61**	**46.2**	**21.43**

**Fig. 3:**
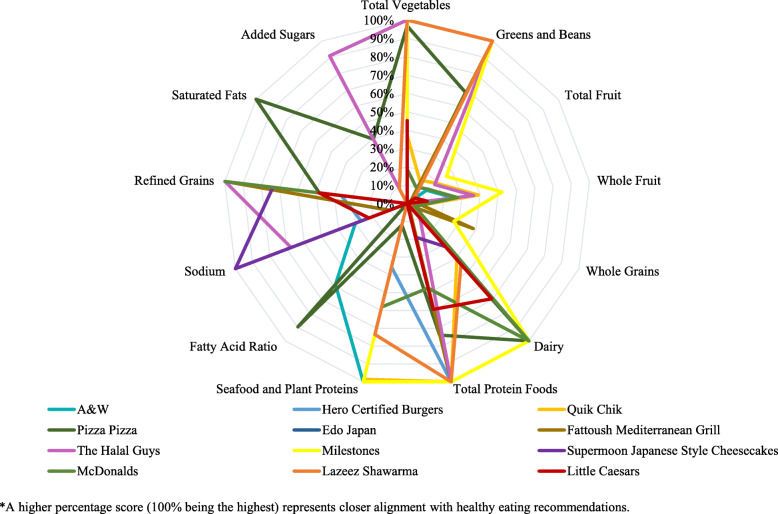
Percent of HEI-2015 component contributing to total HEI-2015 score, by retailer

For the Total Vegetables component 58% of menus received the maximum score. For the Greens and Beans, the Dairy, and the Seafood and Plant Proteins components, 42% of menus received the maximum score. For the Total Protein Foods 67% of the menus received the maximum score. For the Sodium and the Saturated Fats components, only one menu each received the maximum score. None of the menus received the maximum score for the components Total Fruit, Whole Fruit, Whole Grains, Fatty Acid Ratio, and Added Sugars. Figure 3 shows the contribution of HEI-2015 components to each menu’s total HEI-2015 score.

## Discussion

This research establishes an empirical foundation for understanding how OFDS changes geographic access to food in a North American context, and sets the stage for future research to examine how OFDS may ultimately affect dietary health at a population level. We examine delivery distances from 24 randomly selected urban postal codes across Ontario, and used the HEI-2015 to assess diet quality of 12 menu offerings from four popular OFDS apps in a large Canadian city. Given the predicted rapid expansion of OFDS, it is important to consider how OFDS offerings may contribute to population-level nutrition [12]. Briefly, the distance of these retailers from delivery locations across urban Ontario ranged from 0.3 km to 9.4 km, which may have implications for how researchers characterize the retail food environment going forward. Second, perhaps unsurprisingly, menus of the top three retailers available on each of UberEats, DoorDash, SkiptheDishes and Foodora scored poorly in relation to dietary guidance: most retailers’ menus scored fewer than 36 points out of 100. To our knowledge, this paper is the first to examine the healthfulness of foods and beverages currently marketed on OFDS platforms. Below, we review key findings and limitations of this work, and suggest future directions for research.

OFDS (specifically, DoorDash) expanded the retail food environment up to 9 km and up to 472 restaurant choices in this sample of Ontario postal codes. While geographic access is of course not the only determinant of population diet, the convenience of home delivery as well as the plethora of choices may indeed affect FAFH consumption. OFDS delivery ranges are larger than typical telephone delivery because retailers outsource delivery services to OFDS apps, which is more cost effective for retailers, and allows for greater profits, and access to wider markets [19]. Research from China has found increased use of OFDS is associated with an individual’s unwillingness to go out, lack of cooking skills, attraction to sales promotion, bad weather, preference, and habit [10]. Therefore, OFDS availability can interact with personal (e.g., unwillingness to go out or lack of cooking skills) or environmental (e.g., inclement weather) characteristics to increase FAFH access.

On average, distance to retailer was slightly shorter for the first 10 retailers displayed (3.2 km) vs. the last 10 retailers displayed (4.6 km), indicating that distance to retailer may factor into how retailers are displayed in OFDS apps. Distance and time to delivery are not perfectly correlated, and would depend on both physical (built environment) features as well as traffic, but may influence retailer selection from an OFDS platform. Moreover, while this study aims to begin to build a foundation for research on OFDS and dietary health by assessing the extent to which OFDS apps expand retail food environments and the healthfulness of OFDS food offerings, it does not address behavioural aspects of OFDS use. This is an important topic for future research that could be assessed via survey methods of OFDS users. Of particular interest would be how users interact with different OFDS platforms, whether consumers remain loyal to one type of app over time, and reasons for different platform use. In addition, it would be interesting to understand how retailers themselves interact with OFDS platforms, which could be another direction for future research.

As a final point related to our first objective, we found substantial variation in the number of retailers available by postal code even within the same city. Not surprisingly, population size and population density at the city level were relatively strongly correlated with the total number of retailers available in associated postal codes, but the total number of retailers was only weakly associated with mean travel distance for identified retailers. Future research could examine how smaller area contexts (e.g., density of retailers in the physical retail food environment) is associated with the OFDS food environment. Better understanding how physical retail food environments are associated with online food environments may help researchers to more accurately characterize individuals’ exposures to the retail food environment, which is currently a major challenge in extant research [20].

Second, it was not surprising that menus available on OFDS applications scored poorly in terms of diet quality, given prior research evaluating the diet quality of fast food outlets [13] as well as research showing that FAFH consumption is associated with reduced diet quality, higher consumption of energy, fat and saturated fatty acid [21–23]. OFDS consumers, like consumers of brick-and-mortar fast food outlets [13], may be limited in terms of choosing nutritious options. In our sample of retailers, no retailer scored a maximum score for the Total Fruit, Whole Fruit, Whole Grains, Fatty Acid Ratio, and Added Sugars components of the HEI-2015, which indicates current retailers supply energy-dense (high in fat and sugar) foods of low nutritional quality. To the extent that consumer food choices at these retailers align with the nutritional quality of foods typically available, these findings also align with prior research on the poorer nutritional status of individuals who regularly consume FAFH.

### Limitations

This study was among the first to explore OFDS in North America, and has several limitations to take into account. First, to examine how OFDS apps may expand retail food environments, we used data from 24 randomly selected urban postal codes with DoorDash access in Ontario. Unfortunately, postal code data (necessary for app use) is not linked to land use data (e.g., residential vs. commercial vs. industrial) in a publically accessible provincial database. Therefore, we do not know the extent to which OFDS apps may expand retail food environments around homes vs. around work locations. This is an important topic for future research.

Second, while we followed Kirkpatrick and colleagues’ approach to summing all dietary constituents for the entire menu of the retailers included in these analyses, it does not capture the variability in nutritional composition across individual menu items or meal combinations. Therefore, this approach may not be entirely realistic in terms of how it characterizes the nutritional quality of a meal ordered by an OFDS user. Future research should examine the nutritional composition of the most commonly ordered menu items, albeit the proprietary nature of the data may make them difficult to obtain. In addition, the HEI-2015 also does not account for components that are present in excess amounts on menus. For instance, most retailers scored a maximum score in the Total Protein component because a higher score is given if more is present. However, the HEI-2015 does not take into consideration whether these components are present in excess amounts. In the future, points should be taken off if items are present in excess. This limitation is consistent with other studies using the HEI to assess retailer menus [13]. Last, the assumptions made for each retailer (i.e. include all toppings) may not reflect common practices when individuals are actually ordering, which could influence the HEI-2015 score. However, despite these limitations the HEI-2015 tool is an effective tool to use to assess food retailer menus and their alignment to the 2020 Dietary Guidelines for Americans [11, 23].

A third limitation is that we did not assess children’s menus. While many OFDS apps require customers to be at least 18 years of age, this is not an enforceable requirement. Moreover, parents frequently purchase fast food and other FAFH for their families: a recent report by the United States Department of Agriculture’s Economic Research Service [24] found that households with children purchase 19% more fast food meals than households without children, and that single parent-headed households purchase 14% more FAFH relative to other households. Future research (for example, future survey research with OFDS users described above) could examine how different subgroups of the population (e.g., households with children, particularly single-parent-headed households, or people who may have mobility restrictions) use OFDS apps to procure foods. Understanding how diverse groups use OFDS and the dietary implications associated with OFDS use, could also help support equity-focused interventions to support population nutrition among children as well as other vulnerable population subgroups.

Many businesses use internet history and website cookie data to provide more targeted advertisements [25]. While this was out of scope for this research, future research could assess different marketing techniques used by OFDS apps to find opportunities where policy interventions would be beneficial and/or areas where these techniques could be leveraged to support healthier behaviours. A content analysis of OFDS apps could also be conducted to determine the availability of nutrition information on OFDS platforms. Finally, as noted above, conducting surveys with OFDS users would help to elucidate patterns of use of OFDS and further clarify the relationship between physical and online food environments. Given the anticipated growth of OFDS use, future research on the impact of OFDS use on health outcomes is warranted.

## Conclusions and future research

This study offers a glimpse into the OFDS food environment from 2019. The rapidity with which OFDS platforms are growing and changing indicates the dynamic nature of this environment and accentuates the need for surveillance in this area. This is particularly true in the midst of an ongoing global pandemic where OFDS platforms and geographic coverage have been rapidly expanding. OFDS apps may substantially increase geographic access to FAFH sources. There is much room for research on OFDS in North America. As we have outlined above, future research should explore consumer and retailer interactions with and use of OFDS platforms, examine how small area-level contextual factors (e.g., population demographic characteristics) shape OFDS use. Another potentially fruitful avenue of research would be to examine not only dietary outcomes associated with OFDS use, but also other potential health outcomes of OFDS use, such as increased sedentary behaviours and decreased socialization, which may hinder mental health outcomes [10].

## Data Availability

The datasets used and/or analysed during the current study are available from the corresponding author on reasonable request.

## Abbreviations

OFDS: Online Food Delivery Service
FAFH: Food Away From Home
HEI: Healthy Eating Index
USDA: United States Department of Agriculture
FNDDS: Food and Nutrient Database for Dietary Studies
FP: Food Patterns
